# Bibliometric Analysis of Literature in Snake Venom-Related Research Worldwide (1933–2022)

**DOI:** 10.3390/ani12162058

**Published:** 2022-08-12

**Authors:** Fajar Sofyantoro, Donan Satria Yudha, Kenny Lischer, Tri Rini Nuringtyas, Wahyu Aristyaning Putri, Wisnu Ananta Kusuma, Yekti Asih Purwestri, Respati Tri Swasono

**Affiliations:** 1 Faculty of Biology, Universitas Gadjah Mada, Yogyakarta 55281, Indonesia; 2 Faculty of Engineering, University of Indonesia, Jakarta 16424, Indonesia; 3 Research Center for Biotechnology, Universitas Gadjah Mada, Yogyakarta 55281, Indonesia; 4 Department of Computer Science, Faculty of Mathematics and Natural Sciences, IPB University, Bogor 16680, Indonesia; 5 Department of Chemistry, Faculty of Mathematics and Natural Sciences, Universitas Gadjah Mada, Yogyakarta 55281, Indonesia

**Keywords:** snake venom, bibliometry, VOSviewer

## Abstract

**Simple Summary:**

Around the world, snake envenomation poses a serious health risk. Proteins with pharmacological effects are present in snake venom. Recent studies elaborate snake venom and its potential application, including as a cancer drug and antibacterial substances. Our study aimed to analyze the global profile of the literature in snake venom research from documents indexed in the Scopus database between 1933 and 2022. In total, 2999 documents were published with Brazil showing the highest productivity. Antivenom, proteomics, and transcriptomics are emerging as hot topics on a global scale. The present study offers a distinctive overview of snake venom research conducted worldwide.

**Abstract:**

Snake envenomation is a severe economic and health concern affecting countries worldwide. Snake venom carries a wide variety of small peptides and proteins with various immunological and pharmacological properties. A few key research areas related to snake venom, including its applications in treating cancer and eradicating antibiotic-resistant bacteria, have been gaining significant attention in recent years. The goal of the current study was to analyze the global profile of literature in snake venom research. This study presents a bibliometric review of snake venom-related research documents indexed in the Scopus database between 1933 and 2022. The overall number of documents published on a global scale was 2999, with an average annual production of 34 documents. Brazil produced the highest number of documents (*n* = 729), followed by the United States (*n* = 548), Australia (*n* = 240), and Costa Rica (*n* = 235). Since 1963, the number of publications has been steadily increasing globally. At a worldwide level, antivenom, proteomics, and transcriptomics are growing hot issues for research in this field. The current research provides a unique overview of snake venom research at global level from 1933 through 2022, and it may be beneficial in guiding future research.

## 1. Introduction

Venom glands are considered a unique morphological and physiological adaptation developed by animals during evolution to increase the efficacy of capturing prey and as part of a defense system against predators [1,2,3,4,5]. A growing body of research attempting to dissect the composition and possible application of animal venoms has been accumulating for decades [6,7,8,9]. In particular, snake venoms, consisting of various types of proteins and small peptide cocktails, have been gaining significant attention as novel sources of drug discovery in recent years [10,11,12]. The main reason for understanding the compositions of snake venom is that snake bites are considered serious health and economic problems worldwide [13,14,15,16]. Annually, it is reported that more than 2 million snake envenomations occur globally, leading to a high mortality rate in Asia, Africa, and America [17,18,19]. Therefore, snake envenomation was officially classified as a priority neglected tropical disease by the World Health Organization (WHO) in 2017 [20].

Snake venom comprises a vast range of proteins and peptide isoforms, causing a diverse array of immunological and clinical effects when injected [21,22,23]. The secreted phospholipases A2 (PLA2s), snake venom serine proteases (SVSP), three-finger peptides (3FTX), and snake venom metalloproteinases (SVMP) are commonly found enzymes in snake venom. These enzymes alone or in combination have been reported to cause respiratory arrest, inflammation responses, paralysis, necrosis in local tissue, coagulopathy, and hemorrhage upon administration [21,24,25,26,27,28,29,30,31]. Intravenous delivery of antivenom in conjunction with analgesic drugs, hydration treatment, hemodialysis, or supplementation of antibiotics are commonly used to treat snake envenomation [17]. However, the recovery process from snake envenomation has been hampered by the high prevalence of musculoskeletal disabilities and even mortality [32,33,34].

Multiple studies reported various perspectives on snake venom and its applications in the field of medicine and health [11,12,35,36]. Concerns over the swift rise in microbial drug resistance have prompted researchers to explore the promising role of snake venom and its components in eradicating superbugs (Table S1) [37,38,39,40,41,42,43,44,45]. In parallel, numerous studies have also been conducted to isolate and characterize peptides found in snake venoms as potential cancer drugs (Table S2) [46,47,48,49,50,51,52,53,54,55,56,57,58,59,60,61,62,63,64,65,66,67,68,69,70,71,72,73,74,75,76,77,78,79,80,81,82,83,84,85,86,87]. Additionally, in recent years, conjugation of snake venom with monoclonal antibodies has been implemented as a favorable method for designing clinically effective anticancer agents [88]. Recent developments in drug discovery techniques also enable the combination of peptides extracted from snake venom with nanoparticles, allowing customized delivery to the target specific cells or tissues [89,90].

The WHO promotes a periodic review of current development in neglected tropical diseases, including snake bite, to support national and global research capacity [91,92]. Notably, bibliometric analysis has been extensively used to analyze the global research output of various neglected tropical diseases [93,94,95]. However, previous studies in snake venom-related research focused primarily on the clinical application of snake venom, with little attention paid to the progress, current state, and future direction of research in this field. To the best of our knowledge, there are currently no bibliometric studies that qualitatively and quantitatively evaluate the output of snake venom-related research. Therefore, assessing the global research profile of literature on snake venom is critical. This study focused on providing a comprehensive profile of snake venom-related literature for the last eight decades by mapping international collaboration, evaluating the performance of prominent institutions, examining the productivity of prestigious journals, dissecting the characteristics of highly cited articles, and highlighting the emerging research topics. The findings of the current study may provide a visual overview of research progress in this field, as well as assist researchers and practitioners in evaluating the research impacts.

## 2. Materials and Methods

The Scopus database was used to retrieve all snake venom-related documents, excluding erratum, published between 1933 and 2022. Scopus was regarded as the primary source of bibliometric analysis in various disciplines [96,97,98,99]. Using the key terms [“snake” AND “venom”] in the ‘title’ and ‘abstract’ fields, a bibliometric filter to capture snake venom-related publications from the Scopus database was established and performed in May 2022. Type of document, year of publication, institutions, countries, journal titles, citations, and key terms were extracted. The extracted data were analyzed using VOSviewer [100].

## 3. Results

**Figure 1:**
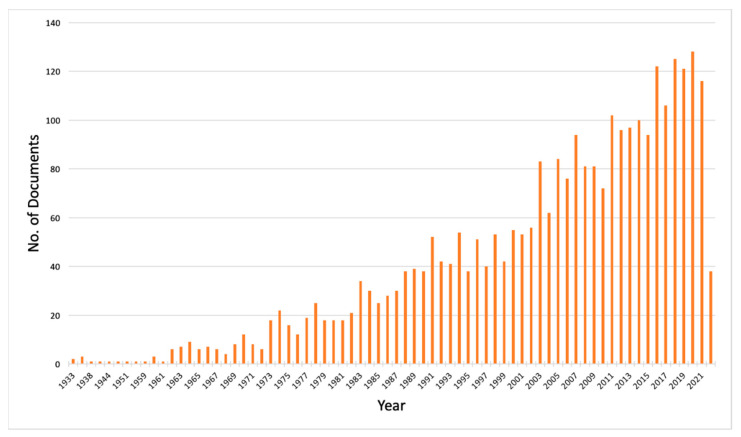
Publication profile of snake venom-related research during the years 1933–2022. A total of 2999 documents were retrieved from the Scopus database. The productivity in snake venom-related research has gradually increased since the 1960s, with the highest number of documents published in 2020.

Between 1933 and 2022, 2999 documents were published globally, resulting in an average annual production of 34 documents related to snake venom. Research articles (*n* = 2629; 87.66%) account for the highest number, followed by reviews (*n* = 268; 8.93%), book chapters (*n* = 46; 1.53%), and conference papers (*n* = 37; 1.23%). The majority of the documents (*n* = 2869; 95.66%) were written in English, followed by Chinese (*n* = 52; 1.73%), Spanish (*n* = 34; 1.13%), and Russian (*n* = 20; 0.66%). Since 1963, the number of snake venom-related documents has gradually increased, with the maximum productivity observed in 2020 (*n* = 128; 4.26%) (Figure 1).

**Figure 2:**
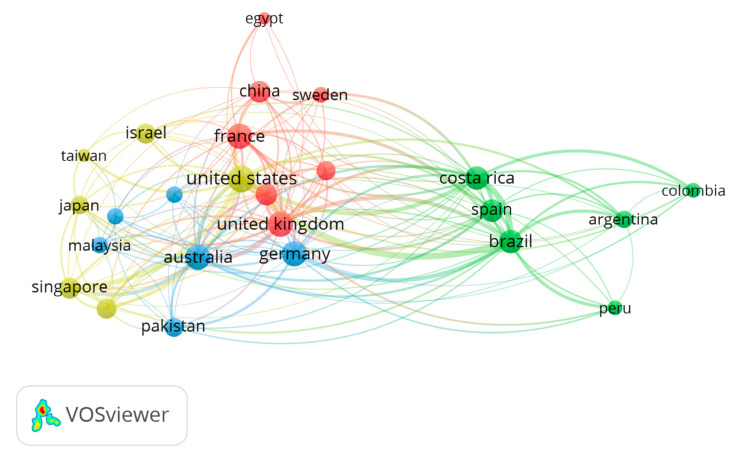
Mapping of country collaboration. Out of 138 countries, 25 published a minimum of 30 documents. The size of the circle is proportional to the number of collaborations with other countries.

Between 1933–2022, 138 countries contributed to the literature on snake venom. The top 10 most productive countries listed a publication share ranging from 24.3% for Brazil to 3.33% for Germany. Table 1 illustrates the top ten countries in terms of their proportionate contribution to the total number of documents on a worldwide scale. Brazil produced the most documents with 729 (24.3%) documents, followed by the United States (*n* = 548; 18.2%), Australia (*n* = 240; 8%), Costa Rica (*n* = 235; 7.83%), and the United Kingdom (*n* = 208; 6.93%). The United States (*n* = 24) listed the highest number of international collaborations, followed by Australia, Germany, and the United Kingdom (*n* = 21) (Table 1; Figure 2).

Table 2 shows the top ten journals with the highest number of documents worldwide, totaling 1082 (36.07%) documents. *Toxicon* (*n* = 682; IF = 2.74), *Toxins* (*n* = 115; IF = 4.086), and *Journal of Venomous Animals and Toxins including Tropical Diseases* (*n* = 47; IF = 2.71) were the most prolific journals on the subject of snake venom. Research articles with the highest number of citations in Table 3 highlight the landmark studies in snake venom-related research and can be used as references in determining the current trends and future directions.

Table 4 shows the global performance of the top 10 productive institutions in the field of snake venom from 1933 to 2022, with a total of 1192 (39.74%) documents. The Universidad de Costa Rica in Costa Rica is the most prolific contributor with 240 (8%) snake venom-related documents. The Instituto Butantan in Brazil (*n* = 228; 7.60%), the Universidade de São Paulo in Brazil (*n* = 213; 7.10%), the Universidade Estadual de Campinas in Brazil (*n* = 95; 3.16%), and the National University of Singapore in Singapore (*n* = 77; 2.56%) were listed second through fifth.

**Figure 3:**
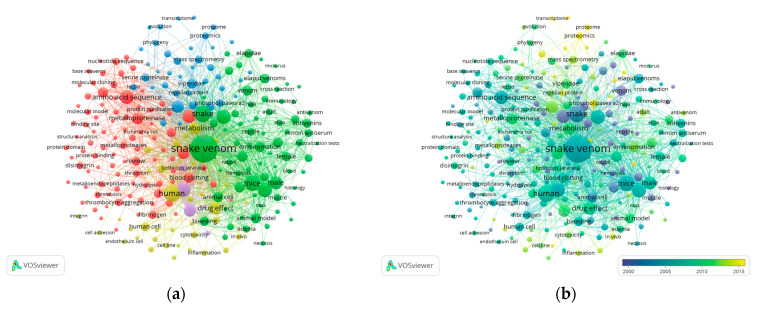
VOSviewer mapping of occurrence terms extracted from titles and abstracts in snake venom-related research articles. (**a**) network visualization; (**b**) overlay visualization. The size of the circles is proportional to the frequency of appearances. The length of the link indicates the degree of relationship. With a minimum of 50 occurrences, 255 out of 15,498 terms match the criteria.

Figure 3 maps the occurrence of terms retrieved from 2999 documents related to snake venom indexed by Scopus. Among the 15,498 extracted terms, 255 were detected to be present in more than 50 occurrences, resulting in 5 distinguished clusters: red, blue, green, yellow, and purple (Figure 3a). Cluster 1 (red color) includes terms such as amino acid sequence, metalloproteinase, blood clotting; cluster 2 (green color): envenomation, animal model, mice; cluster 3 (blue color): viperidae, mass spectrometry, proteomics; cluster 4 (yellow color): drug effect, human cell, metabolism; cluster 5 (purple color): crotalid venoms, bothrops. In Figure 3b, VOSviewer categorizes the extracted terms into a color gradient from blue to yellow, representing old to new publication years. The early years of snake venom-related studies elaborated on several key terms such as drug effect, venom, disintegrin, phospholipase A2, cytotoxicity, and amino acid sequence. Meanwhile, the emerging topics in recent years includes antivenom, proteomics, and transcriptome.

## 4. Discussion

The current study thoroughly examined global research output on the topic of snake venom. According to our findings, snake venom has garnered much interest from scientists all around the world in the last 89 years. The gradual increase in snake venom-related documents since the 1960s could be associated with the funding of the International Society on Toxinology (IST) in 1962 [110,111]. *Toxicon*, the official journal of IST, was listed as the most prolific journal with the highest number of documents related to snake venom in this study. The earliest document from *Toxicon* retrieved in this study was published in 1962 titled “Hemolytic action of indirect lytic snake venom in vivo” by De Vries et al. from Israel [112]. In total, *Toxicon* journal contributed 22.74% (*n* = 682) of the total documents extracted from the Scopus database, indicating the significant impact of *Toxicon* in the development of snake venom-related studies. Interestingly, despite the fact that the oldest article related to snake venom in *Toxins* journal was published in 2009 [113], *Toxins*, by Multidisciplinary Digital Publishing Institute (MDPI), was recognized as the journal with the second highest number of published documents (*n* = 115; 3.83%). The designation of snake bites as a priority neglected tropical disease by WHO in 2017 also affects the growth of documents related to snake venom research [20]. In the period 2017–2021, with an average of 119 documents per year, a total of 596 (19.87%) documents were published, indicating a high research productivity in the field of snake venom in recent years.

Our results showing that Brazil is the most prolific country in terms of snake venom research could be explained by the fact that Brazil is the home to a highly diverse species of snakes [114,115]. Additionally, the high prevalence of snake envenomation in Brazil promotes extensive efforts for prevention and management of snake bites, as well as elaborating the potential application of snake venom in medicine in this country [116,117,118,119,120,121]. Consistent with the result showing that Brazil is the most productive country, Brazil is home to 6 of the 10 institutions with the highest number of documents in snake venom-related research. Other developing countries, such as Costa Rica and India, were among the most productive countries in the field of snake venom research, which could be linked to multiple reports of the snake biting cases in these countries [122,123,124,125,126,127,128]. Our results also demonstrate that developing countries published a relatively high percentage of research articles, indicating that snake venom-related research is not limited to developed countries. Taken together, these findings suggest that the study of snake venom is currently emerging as a global effort.

The number of citations obtained by research articles might be used to determine the central topics in a certain field [129,130,131]. The “Astacins, serralysins, snake venom and matrix metalloproteinases exhibit identical zinc-binding environments (HEXXHXXGXXH and Met-turn) and topologies and should be grouped into a common family, the ‘metzincins’” article by Bode et al., from Germany, published in FEBS Letters, was the most frequently cited article [101]. Importantly, our bibliometric analysis also revealed that snake venom-related articles and reviews were published in reputable journals such as *Nature* and *Science* [105,106,132,133,134].

Up to 2010, researchers reported various studies related to disintegrin, venom, amino acid sequence, phospholipase A2, cytotoxicity, and drug effect. Recent focus on snake venom-related research has been gradually shifting to antivenom, proteomics, and transcriptome, providing hints to the emerging subjects in snake venom-related research in the future. In the last few years, there has been an increase in the publication of snake venom proteomes, especially from the families of Elapidae and Viperidae (Table S3) [135,136,137,138,139,140,141,142,143,144,145,146,147,148,149,150,151,152,153,154,155,156,157,158,159,160,161,162,163,164,165,166,167,168,169,170,171,172,173,174,175,176,177,178,179,180,181,182,183,184,185,186,187,188,189,190,191,192,193,194,195,196,197,198,199,200,201,202,203,204,205,206,207,208,209,210,211,212,213]. To estimate the protein diversity and abundance, characterization of snake venom proteomes involves two main steps: identification of the proteins and peptides followed by quantification [214]. In general, to improve the efficiency of the protein identification step, de-complexing procedures were highly recommended before performing mass spectrometry [215,216,217]. The established protocols generally involve the following workflows: Reverse-Phase High-Performance Liquid Chromatography (RP-HPLC), 1D SDS-PAGE, and in-gel trypsin digestion followed by mass spectrometry (MS) [218,219,220]. The coverage of proteome identification might be improved by incorporating various approaches, including the utilization of venom gland transcriptome libraries and a top-down/bottom-up combination of mass spectrometry [216,221].

In the field of transcriptomics, cloning technology served as the foundation for the early studies of venom gland transcriptome [170,222]. High-throughput RNA sequencing obtained from venom glands is now possible due to the development of next generation sequencing (NGS) technologies [223]. A growing number of venom gland transcriptomes of numerous species of snakes have been constructed (Table S4) [5,136,143,147,148,170,182,189,193,222,224,225,226,227,228,228,229,230,231,232,233,234,235,236,237,238,239,240,241,242,243,244,245,246,247,248,249,250,251,252]. The possibilities of examining various genes are one of the most powerful applications of transcriptomics from the snake venom gland. Through comprehensive profiling, identification of novel protein or peptides in snake venom and interspecies comparison is possible [143,236,237,250,253,254]. Additional transcriptomics studies can also be employed to analyze genetic varieties within snake families [234,247]. Notably, a comprehensive analysis of venom gland transcriptome libraries might help in accelerating the discovery of novel antivenoms.

In general, the widely available antivenoms are produced by continuously exposing animals to sub lethal doses of the snake venom [255,256]. However, the animal-derived antivenoms possess several drawbacks, including contamination of irrelevant antibodies, inadvertent allergic reactions, inefficient production methods, and varied outputs [257,258]. Promising antivenom molecules are listed in Table S5 [259,260,261,262,263,264,265,266,267,268,269,270,271,272,273,274,275,276,277]. Generating monoclonal antibodies against specific enzymes in snake venom has been considered as an alternative strategy for developing antivenoms [259,260,261,262,263,264,265]. Additionally, fragments of recombinant antibodies and nanoparticle have shown effectiveness in inactivating snake venom [272,273,274,275,276,277]. Identifying the inhibitors of venom enzymes is also established as an alternative strategy to design novel antivenoms [266,267,268,269,270,271]. Taken together, the future of antivenom development appears promising with monoclonal antibodies, recombinant fragments, and enzyme inhibitors being proven to be effective in neutralizing snake venoms.

Lastly, the limitation of the current study, similar to previous bibliometric analysis [278,279], is that it excluded documents published in journals not indexed by Scopus.

## 5. Conclusions

The current study presents a comprehensive review of snake venom-related research, spanning nearly eight decades of global literature output. According to our findings, Brazil produced the highest number of documents, followed by the United States, Australia, Costa Rica, and the United Kingdom. Studies in the areas of antivenom, proteomics, and transcriptome are expected to gather a considerable amount of interest in the near future. To summarize, the data offered in this study paints a clear picture of the progress made in the field of snake venom research from 1933 to 2022, and it may be helpful in providing insights for future research.

## Figures and Tables

**Table 1:** Contribution and collaboration of the top ten countries in snake venom publication at a worldwide level from 1933 to 2022.

SCR ^a^	Country	No. of Documents (%)	No. of Collaborating Countries ^b^
1	Brazil	729 (24.3)	18
2	United States	548 (18.2)	24
3	Australia	240 (8.00)	21
4	Costa Rica	235 (7.83)	18
5	United Kingdom	208 (6.93)	21
6	Japan	202 (6.73)	11
7	China	182 (6.06)	15
8	India	180 (6.00)	13
9	Taiwan, China	103 (3.43)	6
10	Germany	100 (3.33)	21

^a^ SCR: standard competition ranking. ^b^ Number of collaborating countries with a minimum threshold of 30 documents.

**Table 2:** The top ten journals in the field of snake venom-related research.

SCR ^a^	Journal Title	No. of Documents (%)	Impact Factor ^b^
1	*Toxicon*	682 (22.74)	2.74
2	*Toxins*	115 (3.83)	4.086
3	*Journal of Venomous Animals and Toxins Including Tropical Diseases*	47 (1.56)	2.71
4	*Journal of Proteomics*	40 (1.33)	4.044
5	*Biochimie*	36 (1.20)	4.079
6	*Journal of Biological Chemistry*	35 (1.16)	5.157
7	*International Journal of Biological Macromolecules*	34 (1.13)	6.953
8	*Archives of Biochemistry and Biophysics*	33 (1.10)	4.013
8	*Biochemical and Biophysical Research Communications*	33 (1.10)	3.575
10	*Biochemistry*	27 (0.90)	3.162

^a^ SCR: Standard competition ranking. If two journals share the same ranking number, a gap is left out in the rankings. ^b^ Clarivate Analytics’ Journal Citation Reports (JCR) 2021 were used to calculate impact factors (IF).

**Table 3:** The highest cited articles on snake venom-related research.

SCR ^a^	Authors	Title	Article Type	Year	Journal Title	No. of Citations
1	Bode et al. [101]	Astacins, serralysins, snake venom and matrix metalloproteinases exhibit identical zinc-binding environments (HEXXHXXGXXH and Met-turn) and topologies and should be grouped into a common family, the ‘metzincins’	Article	1993	*FEBS Letters*	630
2	Markland [102]	Snake venoms and the hemostatic system	Review	1998	*Toxicon*	546
3	Bjarnason and Fox [103]	Hemorrhagic metalloproteinases from snake venoms	Review	1994	*Pharmacology and Therapeutics*	483
4	Theakston and Reid [104]	Development of simple standard assay procedures for the characterization of snake venoms	Article	1983	*Bulletin of the World Health Organization*	482
5	Daltry et al. [105]	Diet and snake venom evolution	Article	1996	*Nature*	477
6	Fry et al. [106]	Early evolution of the venom system in lizards and snakes	Article	2006	*Nature*	423
7	Gutiérrez and Lomonte [107]	Phospholipase A2 myotoxins from Bothrops snake venoms	Review	1995	*Toxicon*	422
8	Gutiérrez and Rucavado [27]	Snake venom metalloproteinases: Their role in the pathogenesis of local tissue damage	Review	2000	*Biochimie*	416
9	Fox and Serrano [108]	Structural considerations of the snake venom metalloproteinases, key members of the M12 reprolysin family of metalloproteinases	Article	2005	*Toxicon*	406
10	Matsui et al. [109]	Snake venom proteases affecting hemostasis and thrombosis	Review	2000	*Biochimica et Biophysica Acta*	359

^a^ SCR: Standard competition ranking.

**Table 4:** The most productive institutions in publications related to snake venom.

SCR ^a^	Institution	Country	No. of Documents (%)
1	Universidad de Costa Rica	Costa Rica	240 (8.00)
2	Instituto Butantan	Brazil	228 (7.60)
3	Universidade de São Paulo	Brazil	213 (7.10)
4	Universidade Estadual de Campinas	Brazil	95 (3.16)
5	National University of Singapore	Singapore	77 (2.56)
6	Universidade Estadual Paulista Júlio de Mesquita Filho	Brazil	76 (2.53)
7	Liverpool School of Tropical Medicine	United Kingdom	73 (2.43)
8	Fundação Oswaldo Cruz	Brazil	72 (2.40)
9	National Taiwan University	Taiwan, China	59 (1.96)
9	Universidade Federal de Uberlândia	Brazil	59 (1.96)

^a^ SCR: Standard competition ranking. If two institutes share the same ranking number, a gap is left out in the rankings.

## Data Availability

Publicly available datasets were analyzed in this study. This data can be found here: http://scopus.com accessed on 30 May 2022.

## Supplementary Materials

The following supporting information can be downloaded at: https://www.mdpi.com/article/10.3390/ani12162058/s1. Table S1. Snake venom components as novel drug candidates to eliminate drug resistant bacteria. Table S2. Anticancer properties of snake venom and its components. Table S3. Elapidae and Viperidae families as representatives of the well-characterized proteomics of snake venoms. Table S4. List of snake species for which transcriptomics libraries are available. Table S5. List of promising antivenom molecules.
