# Supplementary material for: Bibliometric Analysis of Literature in Snake Venom-Related Research Worldwide (1933–2022)
Source: Animals (Basel). 2022 Aug 12;12(16):2058. doi: 10.3390/ani12162058 (PMC9405337; doi:10.3390/ani12162058)
Supplement: Supplementary file 1 [file animals-12-02058-s001.zip › animals-1829183-supplementary.pdf]

Table S1. Snake venom components as novel drug candidates to eliminate drug resistant bacteria.

No.	Bacteria strains	Toxins or enzymes	Snake	References
1	MRSA, MDR *Escherichia coli*, MDR *Pseudomonas aeruginosa*	OH-CATH30 and OH-CM6 (cathelicidins)	*Ophiophagus hannah*	[37]
2	MRSA and VRSA	CATHPb1 (cathelicidin)	*Python bivittatu*	[38]
3	MDR *Acinetobacter baumannii* and MRSA	OH-CATH30 (cathelicidins)	*Ophiophagus hannah*	[39]
4	MRSA	Cath-A (cathelicidin)	*Bungarus fasciatus*	[40]
5	MDR *Burkholderia pseudomallei*	Crotoxin B and daboiatoxin (phospholipase A2)	*Crotalus durissus terrificus, Daboia russelli siamensis*	[41]
6	MDR B. *pseudomallei*	metalloproteinase	*Agkistrodon halys*	[42]
7	MDR B. *pseudomallei*	VipTx-II (phospholipase A2)	*Daboia russelli russelli*	[43]
8	MDR *Mycobacterium tuberculosis*	vgf-1 peptide	*Naja atra*	[44]
9	MRSA	pC-CoaTxII phospholipase A2	*Crotalus oreganus abyssus*	[45]

MRSA: methicillin-resistant *Staphylococcus aureus*; VRSA: vancomycin-resistant *Staphylococcus aureus*; MDR: multi-drug-resistant.

Table S2. Anticancer properties of snake venom and its components.

No.	Cell lines	Toxins or enzymes	Snake	References
**Metastasis inhibition**				
1	human melanoma M24met	contortrostatin (disintegrin)	*Agkistrodon contortrix contortrix*	[46]
2	human breast cancer MDA-MB-435	contortrostatin (disintegrin)	*Agkistrodon contortrix contortrix*	[47]
3	human glioblastoma T98G, U87, A172, and U138	contortrostatin (disintegrin)	*Agkistrodon contortrix contortrix*	[48]
4	human hepato-carcinoma HepG2 and SMMC-7721	agkihpin (arginine esterase)	*Gloydius halys Pallas*	[49]
5	human gingival carcinoma Ca9-22	cardiotoxin III	*Naja naja atra*	[50]
6	human breast cancer MDA-MB-231	Vaa-Dis (disintegrin)	*Vipera ammodytes ammodytes*	[51]
**Induction of apoptosis**				
7	human gastric cancer MKN-45, colorectal	l-amino acid oxidases (LAAO)	*Bothrops leucurus*	[52]

No.	Cell lines	Toxins or enzymes	Snake	References
	cancer HUTU, colon carcinoma RKO			
8	human ovarian cancer CAOV3 and OVCAR3	LAAO	*Crotalus adamanteus*	[1]
9	human colorectal cancer LoVo, HTC-116, and HT-29	SVT	*Echis coloratus*	[54]
10	human leukemia HL60, JURKAT, murine melanoma B16F10	LAAO	*Bothrops atrox*	[55]
11	human prostate cancer PC-3 and DU145	SVT	*Vipera lebetina turanica*	[56]
12	human breast cancer MCF-7	rusvinoxidase (LAAO)	*Daboia russelii russelii*	[57]
13	human colon adenocarcinoma HCT116 and HT-29	SVT	*Vipera lebetina turanica*	[58]
14	human teratocarcinoma PA-1 and human ovarian cancer SK-OV3	SVT	*Vipera lebetina turanica*	[59]
15	human neuroblastoma SK-N-MC and SK-N-SH	SVT	*Vipera lebetina turanica*	[60]
16	human lung cancer A549	ACTX-6 (cytotoxin)	*Agkistrodon acutus*	[61]
17	colorectal adenocarcinoma LS174, HCT116, and HT-29	lebein (disintegrin)	*Macrovipera lebetina*	[62]
18	human leukemia HL-60 and hepatocellular carcinoma HepG2	CR-LAAO	*Calloselasma rhodostoma*	[63]
19	HL-60 and HepG2	BthTX-I (PLA2s)	*Bothrops jararacussu*	[64]
20	HepG2, MCF-7, and DU145	SVT	*Naja naja oxiana*	[65]
21	HCT-8, MDA-MB-231	SVT	*Bitis arietans, C. gasperettii, E. coloratus, and Echis pyramidum*	[66]
22	human endo-cervical carcinoma Ca Ski, C33A, A549, MCF-7	SVT	*Vipera lebetina turanica*	[67]
23	human cervical cancer SiHa and HeLa	SVT	*Bothrops jararaca and Bothrops erythromelas*	[68]
24	MCF7	Phospholipase A2 (PLA2)	*Bungarus fasciatus*	[69]
25	HeLa	Pllans–II (PLA2)	*Porthidium lansbergii lansbergii*	[70]
26	human melanoma SK-MEL-28 and LU-1205	lebein (disintegrin)	*Macrovipera lebetina*	[71]
27	MCF-7, SK-BR-3, and MDA-MB-231	BthTX-I (PLA2)	*Bothrops jararacussu*	[72]
28	MCF-7	SVT	*Cerastes cerastes* and *Vipera lebetina*	[73]

No.	Cell lines	Toxins or enzymes	Snake	References
29	HCT-116, SW-480, and HT-29	LAAO	*Crotalus adamanteus*	[74]
**Cell adhesion inhibition**				
30	human fibrosarcoma HT1080, IGR39, HT29-D4, K562	MVL-PLA2	*Macrovipera lebetina transmediterranea*	[75]
31	human melanoma SK-Mel-28	colombistatin (disintegrin)	*Bothrops colombiensis*	[76]
32	human melanoma A-375 and A-549	tzabcanin (disintegrin)	*Crotalus simus tzabcan*	[77]
		Anti-proliferative activities		
33	MCF-7 and A549	LAAO	*Ophiophagus hannah*	[78]
34	glioblastoma HCB151	L-amino acid oxidase (LAAOcdt)	*Crotalus durissus terrificus*	[79]
35	ovarian carcinoma MDAH 2774	saxatilin (disintegrin)	*Gloydius saxatilis*	[80]
36	Hep2 and HeLa	SVT	*Lapemis curtus*	[81]
37	HeLa	disintegrin	*Echis multisquamatis*	[82]
38	HeLa and Hep2	SVT	*Hydrophis spiralis*	[83]
39	MCF-7 and MDA-MB-231	SVT	*Vipera raddei kurdistanica*	[84]
**Inhibition of angiogenesis**				
40	lung cancer NCI-H460	saxatilin (disintegrin)	*Gloydius saxatilis*	[85]
41	HT-1080 and MDA-MB-231	vixapatin (C-type lectin-protein)	*Vipera xantina palestinae*	[86]
42	MDA-MB-231	bothropoidin (metalloproteinase)	*Bothrops pauloensis*	[87]

Table S3. Elapidae and Viperidae families as representatives of the well-characterized proteomics of snake venoms.

No.	Snake	References
**Elapidae**		
1	*Aipysurus laevis*	[135]
2	*Austrelaps labialis*	[136]
3	*Bungarus caeruleus*	[137]
4	*Bungarus candidus*	[138]
5	*Bungarus fasciatus*	[138,139]
6	*Bungarus multicinctus*	[139,140]
7	*Dendroaspis angusticeps*	[141]
8	*Dendroaspis polylepis*	[142]
9	*Drysdalia coronoides*	[143]
10	*Hydrophis cyanocinctus*	[144]
11	*Hydrophis schistosus*	[145]
12	*Micrurus alleni*	[146]
13	*Micrurus altirostris*	[147]
14	*Micrurus fulvius*	*[148]*
15	*Micrurus mipartitus*	[149]
16	*Micrurus mosquitensis*	[146]
17	*Micrurus nigrocinctus*	[150]
18	*Micrurus tschudii tschudii*	[151]
19	*Naja atra*	[140,152]
20	*Naja haje*	[153]

No.	Snake	References
21	*Naja kaouthia*	[154,155]
22	*Naja katiensis*	[156]
23	*Naja melanoleuca*	[157]
24	*Naja mossambica*	[156]
25	*Naja naja*	[158,159]
26	*Naja nigricollis*	[156]
27	*Naja nubiae*	[156]
28	*Naja pallida*	[156]
29	*Oxyuranus scutellatus*	[160]
30	*Pelamis platura*	[161]
31	*Pseudechis papuanus*	[162]
32	*Toxicocalamus longissimus*	[144]
**Viperidae**		
33	*Agkistrodon contortrix*	[163,164]
34	*Atropoides nummifer*	[165]
35	*Atropoides picadoi*	[165]
35	*Bitis caudalis*	[166]
37	*Bitis gabonica*	[166]
38	*Bitis nasicornis*	[166]
39	*Bothriechis aurifer*	[168]
40	*Bothriechis bicolor*	[168]
41	*Bothriechis lateralis*	[168]
42	*Bothriechis marchi*	[168]
43	*Bothriechis nigroviridis*	[167]
44	*Bothriechis schlegelii*	[168]
45	*Bothriechis thalassinus*	[168]
46	*Bothrocophias campbelli*	[169]
47	*Bothropoides pauloensis*	[170]
48	*Bothrops atrox*	[171–175]
49	*Bothrops ayerbei*	[176]
50	*Bothrops barnetti*	[172]
51	*Bothrops caribbaeus*	[177]
52	*Bothrops colombiensis*	[178]
53	*Bothrops cotiara*	[179]
54	*Bothrops diporus*	[180]
55	*Bothrops erythromelas*	[181]
56	*Bothrops fonsecai*	[179]
57	*Bothrops insularis*	[182]
58	*Bothrops jararaca*	[174,183]
59	*Bothrops lanceolatus*	[177]
60	*Bothrops neuwiedi*	[174]
61	*Bothrops pictus*	[172]
62	*Bothrops pirajai*	[184]
63	*Bothrops punctatus*	[185]
64	*Calloselasma rhodostoma*	[186]
65	*Cerastes cerastes*	[187]
66	*Cerrophidion godmani*	[188]
67	*Crotalus adamanteus*	[189]
68	*Crotalus atrox*	[209]
69	*Crotalus basiliscus*	[190]
70	*Crotalus durissus*	[191,192]
71	*Crotalus horridus*	[193]

No.	Snake	References
72	*Crotalus simus*	[194]
73	*Crotalus tigris*	[210]
74	*Crotalus viridis*	[195]
75	*Daboia russelii*	[196–198]
76	*Gloydius brevicauda*	[199]
77	*Gloydius intermedius*	[200]
78	*Lachesis acrochorda*	[201]
79	*Lachesis melanocephala*	[201]
80	*Lachesis muta*	[211]
81	*Lachesis stenophrys*	[201]
82	*Macrovipera lebetinus*	[212]
83	*Macrovipera mauritanica*	[202]
84	*Ovophis okinavensis*	[203]
85	*Porthidium lansbergii*	[204]
86	*Porthidium nasutum*	[188]
87	*Porthidium ophryomegas*	[188]
88	*Protobothrops flavoviridis*	[203]
89	*Protobothrops mucrosquamatus*	[205]
90	*Sistrurus catenatus*	[213]
91	*Tropidolaemus wagleri*	[206]
92	*Vipera anatolica*	[208]
93	*Vipera nikolskii*	[207]
94	*Vipera orlovi*	[207]
95	*Vipera renardi*	[207]

Table S4. List of snake species for which transcriptomics libraries are available.

No.	Snake	References
**Colubridae**		
1	*Dispholidus typus*	[5,224]
2	*Liophis miliaris*	[224]
3	*Liophis poecilogyrus*	[5,224]
4	*Philodryas olfersii*	*[5,224,225]*
5	*Rhabdophis tigrinus*	[5,224]
6	*Telescopus dhara*	[5,224]
7	*Thamnodynastes strigatus*	[226]
8	*Thrasops jacksonii*	[5]
9	*Trimorphodon biscutatus*	[5,224]
**Elapidae**		
10	*Austrelaps labialis*	[136]
11	*Bungarus flaviceps*	[227]
12	*Bungarus multicinctus*	[228]
13	*Demansia vestigiata*	[229]
14	*Drysdalia coronoides*	[143]
15	*Hydrophis curtus*	[230]
16	*Micropechis ikaheca*	[231]
17	*Micrurus altirostris*	[147]
18	*Micrurus corallinus*	[147,232]
19	*Micrurus fulvius*	[148]
20	*Micrurus mipartitus*	[233]

No.	Snake	References
21	*Naja atra*	[228]
22	*Oxyuranus microlepidotus*	[5]
**Homalopsidae**		
23	*Cerberus rynchops*	[234]
24	*Enhydris polylepis*	[224]
**Viperidae**		
25	*Agkistrodon piscivorus*	[235]
26	*Atropoides mexicanus*	[236]
27	*Atropoides picadoi*	[236]
28	*Azemiops feae*	[5,224]
29	*Bitis gabonica*	[237]
30	*Bothriechis lateralis*	[236]
31	*Bothriechis schlegelii*	[236]
32	*Bothropoides pauloensis*	[170]
33	*Bothrops alternatus*	[238]
34	*Bothrops asper*	[236]
35	*Bothrops atrox*	[239]
36	*Bothrops insularis*	[182,222]
37	*Bothrops jararaca*	[240,241]
38	*Causus rhombeatus*	[5]
39	*Cerrophidion godmani*	[236]
40	*Crotalus adamanteus*	[189,242]
41	*Crotalus durissus*	[243,244]
42	*Crotalus horridus*	[193]
43	*Crotalus simus*	[236,245]
44	*Deinagkistrodon acutus*	[246]
45	*Echis carinatus*	[247]
46	*Echis coloratus*	[247]
47	*Echis ocellatus*	[248,249]
48	*Echis pyramidum*	[247]
49	*Lachesis muta*	[250]
50	*Sistrurus catenatus*	[251]
51	*Vipera ammodytes*	[252]

Table S5. List of promising antivenom molecules.

No.	Antivenom molecules	Toxins or enzymes	Snake	References
**Monoclonal antibody (MA)**				
1	MA	toxin alpha	*Naja nigricollis*	[259]
2	MA	metalloproteinase HR1a	*Protobothrops flavoviridi*	[260]
3	MA	dendrotoxins	*Dendroaspis polylepis*	[261]
4	MA	crotoxin	*Crotalus durissus terrificus*	[262]
5	MA	Phospholipases A2	*Bothrops jararacussu*	[263]
6	MA	alpha-neurotoxin	*Naja kaouthia*	[264]
7	MA	unspecified	*Bothrops jararacussu, Crotalus durissus*	[265]
**Small molecule inhibitors**				
8	varespladib (LY315920)	phospholipase A2	*Micrurus fulvius, Vipera berus*	[266]
9	LY333013	phospholipase A2	*Oxyuranus scutellatus*	[267]
10	batimastat and marimastat	metalloproteinase	*Echis ocellatus*	[268]

No.	Antivenom molecules	Toxins or enzymes	Snake	References
11	varespladib	phospholipase A2	*Deinagkistrodon acutus, Agkistrodon halys, Bungarus multicinctus,* and *Naja atra*	[269]
12	batimastat	metalloproteinases	*Bothrops asper*	[270]
13	thiosemicarbazones	metalloproteinases	*Bothrops pauloensis*	[271]
**Nanoparticles**				
14	C60 fullerene	unspecified	*Crotalus oreganushelleri*	[272]
**Antibody fragments**				
15	llama single-domain antibodies VHHs (C2 and C20) and VHH-Fc	α-cobratoxin	*Naja kaouthia*	[273]
16	humanized-single domain antibodies VH/VHH	phospholipase A2	*Naja kaouthia*	[274]
17	camelid Single-Domain Antibodies (VHHs)	crotoxin	*Crotalus durissus terrificus*	[275]
18	recombinant single chain antibody, scFv	metalloproteinase	*Bothrops asper*	[276]
19	single-chain variable fragment (scFv)	unspecified	*Agkistrodon acutus*	[277]
